# Dexamethasone enhances risk of herpes zoster in severe COVID-19 infection

**DOI:** 10.1007/s15010-021-01747-x

**Published:** 2021-12-22

**Authors:** Nathan B. Price, Charles Grose

**Affiliations:** 1 Division of Infectious Diseases, Department of Pediatrics, University of Arizona, Tucson, AZ USA; 2 Division of Infectious Diseases/Virology Laboratory, Department of Pediatrics, University of Iowa Hospital, Room BT2001, 200 Hawkins Drive, Iowa City, IA 52242 USA

We read the review article about herpes zoster and COVID-19 infection with great interest [1]. As an example from the review, the author noted that the Brazilian Ministry of Health had documented an increase in cases of herpes zoster from 9.69 to 14.13 cases per million people in the Rio de Janeiro region pre-and post-COVID-19 epidemic. We propose that we can answer the question whether this association is a coincidence or a causal relationship, at least in several of the severe treated cases. There is a 70-year history in the medical literature about the induction of herpes zoster in a wide variety of patients who were prescribed corticosteroid therapy. We have recently reviewed this association [2]. This association has been documented in both immunocompetent and immunodeficient children and both immunocompetent and immunodeficient adults [3].

Dexamethasone has become commonplace in the treatment protocols for management of more severe cases of COVID-19 infection [4]. A standard dexamethasone dosage is 6 mg per day for 10 days. The consensus in the virology literature is that a daily prednisone dosage of 20 mg enhances the likelihood of herpes zoster in any adult [2]. The dexamethasone/prednisone equivalence between a daily dexamethasone dosage of 6 mg is approximately 37 mg of prednisone, almost twice the known risk dosage of 20 mg. Even smaller dosages of prednisone (7. 5 mg daily, equivalent to ~ 1 mg dexamethasone) have been associated with increased herpes zoster in immunocompromised adults [2, 3]. Furthermore, prednisone has a half-life around 24 h whereas dexamethasone has a longer half-life. Thus, dexamethasone given on a daily basis may accumulate to higher levels over the usual COVID-19 treatment period of 10 days.

When we performed a search on the PubMed website, looking for articles on dexamethasone treatment of COVID-19, we retrieved 788 titles. A brief survey of these articles included an estimation that during 2020, as many as 50% of patients with severe COVID-19 disease received corticosteroids. For example, in a large controlled open-label trial carried out in the United Kingdom by the Recover Collaborative Group, the investigators concluded that the use of dexamethasone resulted in a lower 28-day mortality among COVID-19 patients who were receiving either invasive mechanical ventilation or oxygen alone at randomization [5]. Finally, the guidelines of the Infectious Disease Society of America also recommend dexamethasone therapy for critically ill and severe but not critically ill cases of COVID-19 infection.

As cited in the review [1], most patients developed herpes zoster between 1 to 4 weeks after the diagnosis of COVID-19 infection, the average being 17 days. One case occurred 2 days before diagnosis and another case occurred on the same day of diagnosis. Obviously, herpes zoster cases that occurred before or at the time of diagnosis of COVID-19 infection cannot be caused by dexamethasone, but the evidence in the medical literature strongly suggests that cases of herpes zoster that occur between 1 and 8 weeks after corticosteroid therapy can be secondary to the lymphopenia caused by corticosteroids [2, 3]. To define a comprehensive hypothesis, we propose that COVID-19 infection by itself can on some occasions cause sufficient lymphopenia to lead to herpes zoster before onset of clinical COVID-19 infection. But more commonly we propose that the additive effect of dexamethasone-induced lymphopenia on COVID-19-induced lymphopenia leads to the increased rate of herpes zoster after diagnosis and treatment of COVID-19 infection. Finally, we note that the majority of herpes zoster cases occurred in COVID-19 patients who were older than 50 years and therefore already at higher risk to develop herpes zoster.
